# Acute Multifocal Nonhematogenous Methicillin-Sensitive Staphylococcus aureus Osteomyelitis in a Healthy Adolescent: An Atypical Presentation

**DOI:** 10.7759/cureus.22453

**Published:** 2022-02-21

**Authors:** Radhika Maddali, Esra Fakioglu, Karim Masrouha, Lily Q Lew

**Affiliations:** 1 Department of Pediatrics, Flushing Hospital Medical Center, Flushing, USA; 2 Department of Orthopedic Surgery, New York University Langone Health, New York, USA

**Keywords:** osteomyelitis, mssa, non-hematogenous, multifocal, acute

## Abstract

Osteomyelitis represents inflammation and infection of bone tissue by a pathogen. Acute osteomyelitis is more likely to be unifocal compared to a chronic process which tends to be multifocal and recurrent. Early diagnosis, aggressive appropriate antibiotic therapy and a multidisciplinary approach are essential for a satisfactory prognosis and improved outcome. We report an atypical case of acute multifocal methicillin-sensitive *Staphylococcus aureus *(MSSA) osteomyelitis.

## Introduction

Inflammation and infection of bone tissue, known as osteomyelitis, occur infrequently due to the nature of bone. Extracellular and cellular matrix of bone are generally resistant to entrance and proliferation of microorganisms excluding extremes of ages [1]. Early recognition of symptoms, rapid diagnosis and aggressive antibiotic therapy result in favorable sequelae and prevention of chronic recurring infection [1]. Obtaining appropriate cultures, imaging studies and inflammatory markers can facilitate the diagnosis and management. In addition to aggressive prolonged antibiotic therapy, a multidisciplinary approach contributes to optimal recovery.

The incidence of skateboard-related injuries as tracked by the National Trauma Data Bank has increased in recent years [2]. Injuries to the extremities occur frequently despite proper use of safety gear. Healthcare providers need to be vigilant of the potential development of osteomyelitis after minor trauma. We report a case of a previously healthy adolescent with acute multifocal nonhematogenous methicillin-sensitive *Staphylococcus aureus* (MSSA) osteomyelitis after a skateboard accident.

## Case presentation

A previously healthy 12-year-old Hispanic male presented with a nine-day history of left ankle pain after a skateboard accident and a two-day history of right wrist pain with limited range of motion. He experienced subjective fever in the preceding five days that was managed with acetaminophen. Two days after the accident, he was discharged from an emergency department with the diagnosis of ankle sprain after having been evaluated by physical examination and obtaining a negative plain radiograph. There was no history of recurrent skin infection, prior arthralgia or arthritis, animal contact nor was there recent domestic or international travel. He was not sexually active and was up to date with his vaccinations. On examination, he weighed 48.1 kg, had a temperature of 38.3°C, a blood pressure of 106/69 mmHg and pulse of 101 beats per minute. The head and neck examinations were normal. There was no lymphadenopathy. Heart sounds were normal, and rhythm was regular on auscultation. The left ankle and right wrist were swollen with warmth and exhibited limited range of motion and diffuse tenderness. The overlying skin of both joints was not erythematous or discolored. Superficial abrasions on the lateral aspect of the left ankle were healing.

Initial evaluation included plain radiograph, culture of left ankle skin abrasions and blood sampling for complete blood count, culture, C-reactive protein (CRP) and erythrocyte sedimentation rate (ESR). The patient was started on a broad-spectrum empiric antibiotic regimen consisting of vancomycin (80 mg/kg/day intravenously divided every six hours) and ceftriaxone (75 mg/kg/day intravenously divided every 12 hours) to cover both MSSA and community-acquired methicillin-resistant *Staphylococcus aureus* (CA-MRSA).

Hospital course

**Figure 1:**
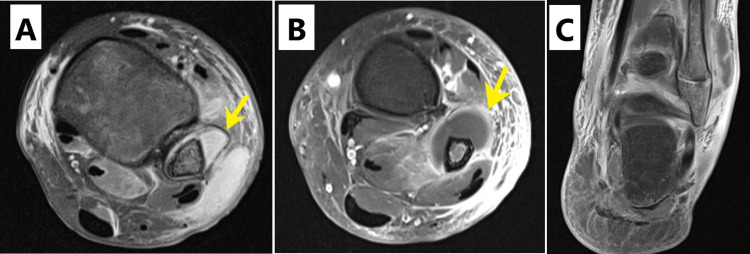
Magnetic resonance imaging (MRI) of the left ankle. (A) Axial proton density fat saturation view demonstrating a subcutaneous and subperiosteal distal fibula abscess (yellow arrow) and surrounding subcutaneous edema. (B) Axial T1-weighted fat saturation view demonstrating the same abscess with peripheral enhancement (yellow arrow) postgadolinium contrast and surrounding subcutaneous edema. (C) Coronal T1-weighted fat saturation, postgadolinium contrast demonstrating subperiosteal abscess at the level of lateral malleolus with surrounding subcutaneous edema.

**Figure 2:**
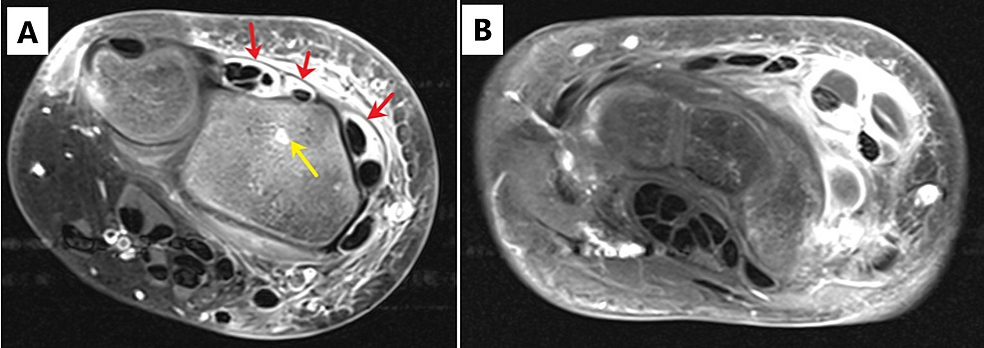
Magnetic resonance imaging (MRI) of the right wrist. (A and B) Axial T1-weighted fat saturation, postgadolinium contrast demonstrating fluid in multiple extensor tendon sheaths (red arrows) with surrounding edema, suggestive of pyogenic extensor tenosynovitis. There was also enhancement within the distal radius, suggestive of possible osteomyelitis (yellow arrow).

**Figure 3:**
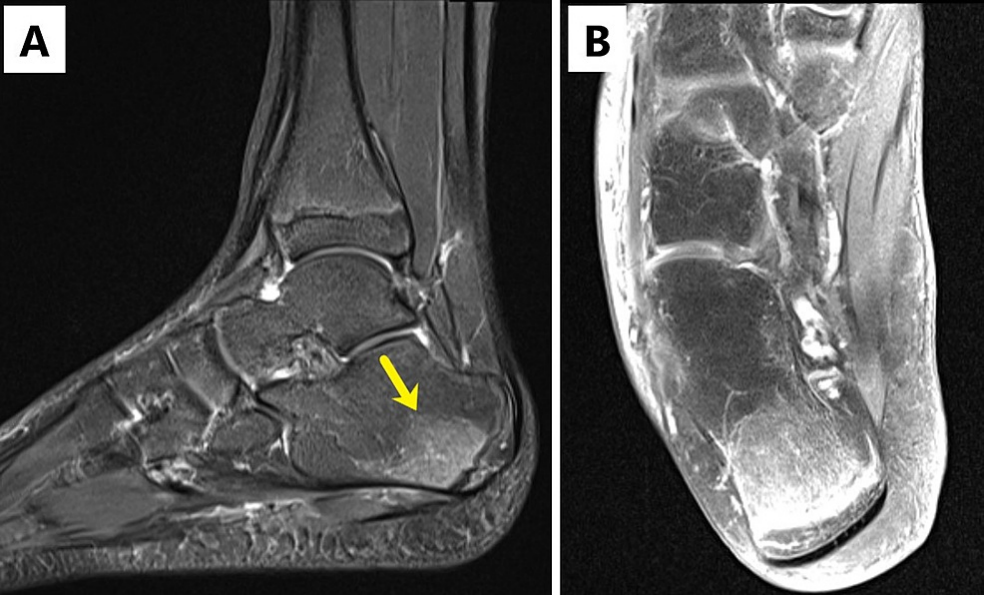
Magnetic resonance imaging (MRI) of the right ankle and foot. (A) Sagittal inversion recovery demonstrating increased signal intensity within the calcaneus (yellow arrow). (B) Coronal T1-weighted fat saturation, postgadolinium contrast demonstrating enhancement in the area of concern, suggestive of osteomyelitis.

Plain radiographs of left ankle and right wrist were negative for fracture, bone lesions and periosteal elevation. Magnetic resonance imaging (MRI) of both ankles and right wrist was obtained for further evaluation. The studies demonstrated marrow edema, postgadolinium enhancement of the distal fibula, metaphysis and epiphysis and a lobulated complex fluid collection adjacent to distal fibula of the left ankle (Figures 1A-1C) along with marrow edema of the right distal radius and extensor compartment tenosynovitis (Figures 2A, 2B), as well as enhancement within the calcaneus of the right foot (Figures 3A, 3B). Culture of left ankle superficial abrasions grew MSSA. The patient underwent drainage, irrigation and debridement of the left distal fibula as well as right wrist extensor tendon tenosynovitis. Purulent fluid from both sites was submitted for Gram stain, aerobic culture, fungal culture and acid-fast bacilli. Antibiotic therapy was changed to nafcillin (200 mg/kg/day intravenously divided every six hours) when both wound and abscess cultures grew MSSA sensitive to penicillin and clindamycin. On the third hospital day, a new right ankle swelling was identified. Ultrasound of the swelling identified a 3 cm collection of fluid. Systemic antibiotic treatment continued for one week before transitioning to oral cephalexin (50-100 mg/kg/day in three divided doses) based on susceptibility pattern for a total of six weeks. Echocardiogram did not show intracardiac vegetation. Blood cultures remained negative. Sequential monitoring of inflammatory markers demonstrated a downward trend. Table 1 shows laboratory and microbiological results. Rehabilitation was provided by occupation and physical therapists. The patient was discharged home on the eighth hospital day with instructions for follow-up with his pediatrician, pediatric infectious disease specialist and pediatric orthopedic surgeon. He showed evidence of wound healing as well as resolution of his swelling and pain. He continued to be asymptomatic at one-year follow-up.

**Table 1:** Results of laboratory and microbiological studies. GAS: Group A beta-hemolytic streptococcus, MSSA: methicillin-sensitive *Staphylococcus aureus.*

Test (SI units)	Day#1	Day#3	Day#7	Reference range
White blood cell count (10^9^/L)	12.6	7.6	9.6	4.8-10.8
Hemoglobin (g/L)	123	111	116	120-160
Platelet count (10^9^/L)	344	444	554	165-332
C-reactive protein (mg/L)	219.0	157.0	18.0	<10.0
Erythrocyte sedimentation rate (mm/hr)	75	71		0-15
Immunoglobulin, total IgG (g/L)			12.9	7.0-16.0
Immunoglobulin A (g/L)			2.3	0.7-4.0
Immunoglobulin M (g/L)			1.02	0.40-2.30
C4 complement (g/L)			0.33	0.14-0.44
C3 complement (g/L)			1.65	0.88-1.65
Throat culture for GAS	Negative			Negative
Streptococcus group antigen screen	Negative			Negative
Antistreptolysin O (IU/mL)	200			0-200
Anti-DNase B (U/mL)		266		<376
Blood culture	No growth	No growth	No growth	No growth
Culture: Left ankle wound	MSSA			No growth
Culture: Left distal fibula abscess				
Aerobic culture	MSSA			No growth
Fungal culture	Negative			Negative
Acid-fast bacilli	Negative			Negative
Gram stain: wound and abscess	Gram-positive cocci in clusters			Nondetected
Potassium hydroxide (KOH) prep test	Negative			Negative
*Neisseria gonorrhoeae* culture	Negative			Negative
Diphtheria antitoxoid antibodies (IU/mL)			2.57	<0.10
Tetanus antibodies (IU/mL)			1.97	<0.15

## Discussion

Osteomyelitis represents inflammation of bone tissue due to infection, commonly by bacteria, and rarely by fungi, mycobacteria or even viruses [1,3]. Despite advances in diagnosis and management, there continues to be substantial mortality and morbidity associated with this condition. The incidence of osteomyelitis in children is 13 cases per 100,000 person-years compared to a higher incidence in adults of 90 cases per 100,000 person-years [4]. Risk factors for osteomyelitis include diabetes mellitus with vascular insufficiency, immune-compromised status, sickle cell disease and intravenous drug abuse [5]. The usual symptoms of osteomyelitis include pain, swelling with warmth, erythema and limited range of motion of involved area and fever [6]. Duration of symptoms defines acuity or chronicity with acute infection being detected within two weeks of symptoms. Symptoms for more than two weeks to within one to several months are seen in both subacute and chronic infections. Constitutional symptoms are usually absent in chronic infection [3]. Alternatively, histopathological findings rather than duration of symptoms may be used to categorize osteomyelitis as acute or chronic. Using this approach, acute osteomyelitis occurs before the development of necrotic bone or sequestra [7]. Our patient’s duration of symptoms and absence of observed necrotic bone were consistent with acute osteomyelitis.

Infectious organisms spread by three major pathways: hematogenous route, contiguous spread after trauma and direct inoculation from surgery [1,7]. Hematogenous spread represents the most common mode of bacterial seeding in multifocal osteomyelitis especially among neonates (50%) compared to older children (8%-9%) [8,9]. Contiguous spread and direct inoculation typically lead to a single focus infection. Additional sources of infection include artificial grass abrasion and body shaving [10].

Acute osteomyelitis commonly involves a single focus (95%) [8]. In contrast, multifocal and symmetrical infections characterize chronic and recurrent infection. Different bone involvement occurs over one to three days, and long bones (75%-90%) of both upper and lower extremities are more likely to be involved [3,8,11]. Growing bones with changing vascularity and anatomy in children and adolescents affect the pathogenesis of osteomyelitis. The metaphysis of long growing bones is frequent destinations for deposition and growth of microorganisms as they travel through the nutrient artery. During growth, the metaphysis is well perfused and yet has a limited number of functioning phagocytes and slow blood flow in vascular loops [9,11].

The most common causative pathogen of osteomyelitis is *Staphylococcus aureus*, a skin organism. The frequency of CA-MRSA isolates matches that of MSSA isolates [12]. Healthy intact bone tends to resist infection [3]. Disruption of bone homeostasis occurs in presence of a pathogen. A specific pathogenic property of staphylococcus species is its ability to adhere to collagen mediated by collagen-binding adhesion, a mosaic protein [13]. Following adhesion, replication of bacteria initiates the pathogenic process. Focal bone necrosis occurs with blood vessel compression secondary to accumulation of exudate under pressure. Panton-Valentine leukocidin (PVL) producing *Staphylococcus aureus* has been described in children with osteomyelitis associated with venous thrombosis [14]. Studies have shown PVL-positive *Staphylococcus aureus* linked to CA-MRSA and multifocal osteomyelitis [15]. Other pathogens besides *Staphylococcus aureus* often isolated in children over age five years include Group A streptococcus and *Neisseria gonorrhea* [16].

Our otherwise healthy immunocompetent adolescent patient had no known risk factors for osteomyelitis and no prior history of recurrent bone infection. Suspicion for nonbacterial inflammatory bone disease and chronic recurrent multifocal osteomyelitis mimicking subacute osteomyelitis was considered despite the absence of autoimmune disease [17]. The injury to his left ankle displayed superficial skin disruption that could have served as a portal of entry. The patient was hospitalized after complaint of pain of a second focus. Multifocal osteomyelitis was demonstrated on MRI. Multiple negative blood cultures and normal echocardiogram to assess for a potential cardiovascular focus excluded hematogenous spread. Superficial wounds and abscess cultures yielded growth of MSSA. Bacteria seeding was likely to occur in the preceding five days of presentation when he had intermittent fever. Positivity rate of blood culture in acute hematogenous osteomyelitis in children and adolescents is less than 50% [18]. We postulate that the mode of spread occurred from the contiguous soft tissue wound with transient bacteremia accounting for negative blood cultures obtained during hospitalization. Rare cases of severe staphylococcal sepsis in otherwise healthy adolescents resulting in multifocal osteomyelitis have been linked to CA-MRSA that encodes gene for PVL and USA300 strain of MRSA [14]. Unfortunately, we could not determine the genetic encoding of this patient’s isolate, and although it was MSSA, it could have possessed the PVL gene for pathogenesis.

Early recognition of symptoms, rapid diagnosis and aggressive antibiotic therapy are associated with excellent outcome and prevention of chronic recurring infection. Our patient presented with symptoms classic for osteomyelitis. Laboratory findings seen in osteomyelitis include leukocytosis and elevated inflammatory markers [19]. He did have leukocytosis but not anemia and thrombocytopenia, thus excluding leukemia and low immunity [8]. Both CRP and ESR were significantly elevated. Plain radiograph, MRI and ultrasound were included in the evaluation. Plain radiograph excluded a fracture and revealed soft tissue swelling. Ultrasound was useful in detecting subperiosteal abscess. MRI was the imaging study of choice for its highest sensitivity and specificity in detecting osteomyelitis [20]. Drainage, irrigation and debridement of subperiosteal abscess of the left distal fibula and the right distal radius not only evacuated the collection but also provided specimen to identify the causative pathogen and its antibiotic susceptibility. The identification of the causative pathogen and susceptibility pattern from purulent fluid was important to guide antibiotic therapy. Detection of Gram-positive cocci in clusters on Gram stain and growth of MSSA sensitive to penicillin confirmed the pathogen. Histopathologic diagnosis was also confirmed by surgical intervention. Fungal and mycobacterium cultures remained negative. Surgical intervention, prolonged appropriate antibiotic therapy and rehabilitation contributed to our patient’s full recovery.

## Conclusions

We conclude that this case demonstrated acute multifocal nonhematogenous MSSA osteomyelitis spread by contiguous soft tissue wound with probable transient bacteremia not identified by blood culture. Recent increasing popularity of skateboarding as a form of recreation and sport is associated with new set of traumatic injuries. Healthcare providers must have a high index of suspicion for osteomyelitis, provide counseling and suggest follow-up in one week especially with persistence of symptoms. This case illustrated an atypical presentation of osteomyelitis and how a rapid diagnosis, aggressive appropriate antibiotic therapy and multidisciplinary approach lead to a full recovery.
